# Prometheus revisited: liver homeostasis and repair

**DOI:** 10.18632/aging.102957

**Published:** 2020-03-23

**Authors:** Tianliang Sun, Stefano Annunziato, Jan S. Tchorz

**Affiliations:** 1 Novartis Institutes for BioMedical Research, Novartis Pharma AG, Basel, Switzerland

**Keywords:** liver homeostasis, liver stem cell, AXIN2, LGR5, SOX9, liver regeneration

The liver is a bioreactor responsible for metabolism of nutrients and xenobiotics, as well as a factory and recycling station for a large number of systemic proteins. Its unmatched regenerative capacity was already described in the myth of Prometheus 2500 years ago and extensively studied over the past decades. However, the exact mechanisms enabling maintenance of liver mass during homeostasis and repair remained unclear. Diploid glutamine synthetase (GS)+ pericentral hepatocytes expressing the WNT/β-Catenin target gene *Axin2* showed superior proliferative capacity over mostly polyploid hepatocytes in other zones. These GS+/AXIN2+ pericentral hepatocytes were considered liver stem cells as they self-renewed and replaced all hepatocytes along the liver lobule during homeostatic renewal [1]. In contrast, lineage tracing using *Lgr5*, another WNT/β-Catenin target gene and pericentral hepatocyte marker, did not support increased proliferative capacity and expansion of pericentral hepatocytes into other zones [2,3]. Likewise, mTERT lineage tracing suggested no zonal dominance of pericentral hepatocytes during liver homeostasis but proposed self-renewing mTERT^high^ hepatocytes with increased proliferative capacity as liver stem cells [4]. Moreover, SOX9+ periportal hepatocytes were described as hybrid hepatocytes with liver stem cell properties as they displayed increased regenerative potential in response to injury [5]. The controversy around the elusive liver stem cell arises from the diverse hepatocyte populations that have been proposed to harbor increased regenerative capacity and the conflicting results originating from different lineage tracing experiments under different housing conditions [1–5]

To exclude the possibility that the heterozygous gene deletion of the lineage tracing marker, as present in previous studies [1,2,4], may bias the proliferative capacity of hepatocytes, we generated a BAC-transgenic AXIN2 lineage tracing model not interfering with WNT/β-Catenin signaling and shed new light on the debated role of pericentral hepatocytes during homeostasis and repair [6]. While we confirmed expansion of AXIN2+ tissue stem cells in stomach, small intestine and colon, we did not observe evidence of AXIN2+ hepatocytes being bona fide liver stem cells. During homeostasis, AXIN2+ hepatocytes neither overtly proliferated nor did they stream and replace hepatocytes in all liver zones [6]. Expression profiling of AXIN2+ versus AXIN2- hepatocytes confirmed expected differences in their metabolic profile but not in their proliferative status. Interestingly, intestinal crypt stem cells showed a strong proliferative gene expression signature when compared to AXIN2+ hepatocytes, whereas they did not express WNT/β-Catenin-controlled metabolic enzymes. Further studies are needed to understand how these two cell types differentially utilize WNT/β-Catenin signaling. Moreover, it remains elusive why pericentral hepatocytes do not overtly proliferate during homeostasis despite their constant WNT/β-Catenin pathway activity necessary to maintain metabolic liver zonation. Since hepatocellular carcinoma often displays overactivated WNT/β-Catenin signaling, the pericentral niche likely requires a robust safeguarding mechanism restricting proliferation that remains to be identified.

In response to partial hepatectomy, an injury model without zonal bias, hepatocytes in all liver zones proliferated equally, without superior regenerative capacity of AXIN2+ pericentral hepatocytes. Interestingly, AXIN2 was upregulated in hepatocytes of all zones prior to initiation of their proliferative response [6], and liver regrowth was significantly impaired upon abrogation of WNT/β-Catenin signaling [2]. Likewise, allyl-alcohol-induced damage of periportal hepatocytes resulted in upregulation of AXIN2 in periportal and adjacent parenchymal hepatocytes that enabled liver repair, with AXIN2+ pericentral hepatocytes even contributing less when compared to hepatocytes in proximity to the injury site [6]. Finally, ablation of the GS+/AXIN2+ pericentral hepatocyte pool only transiently disrupted metabolic zonation and liver homeostasis and stimulated hepatocytes in other zones to proliferate and convert into GS+/AXIN2+ pericentral hepatocytes. Collectively, our data supports that upregulation of AXIN2 in hepatocytes of all liver zones, rather than a pre-existing pericentral AXIN2+ putative hepatocyte stem cell population, enables liver regeneration [6]. Future studies are required to determine the instructive mechanisms which promote spatially confined upregulation of WNT/RSPO ligands to induce WNT/β-Catenin signaling in regenerative hepatocytes. In addition, profiling of hepatocytes which upregulate AXIN2 and re-enter the cell cycle in response to injury, may highlight mechanisms restricting proliferation of AXIN2+ pericentral hepatocytes during homeostasis.

**Figure 1:**
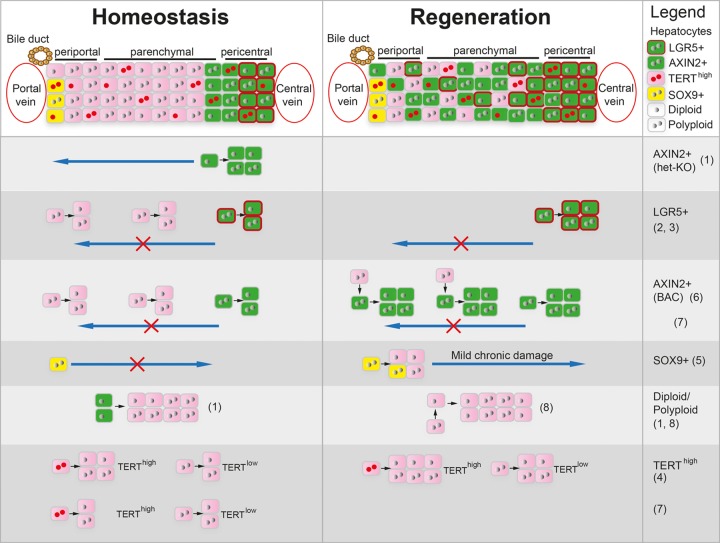
**Different hepatocyte populations proposed to enable liver homeostasis and regeneration.** Scheme depicting hepatocyte populations in the 3 liver zones and their proliferative capacity during homeostasis and regeneration described by different referenced studies. Reference limit did not allow inclusion of all related studies.

Back-to-back with our study, two other reports provided important insights into clonal expansion of hepatocytes in different zones and their plasticity during homeostasis and repair [7,8]. Using AAV-induced random confetti lineage tracing, Holger Willenbring`s group showed that proliferation of hepatocytes in all zones maintains the hepatocyte mass during homeostasis and enables liver repair in response to various insults [7]. Markus Grompe`s team clarified that polyploid hepatocytes have extensive regenerative capacity using a multicolor reporter allele system to genetically label and trace polyploid cells. Polyploid hepatocytes in all liver zones undergo ploidy reduction, proliferation, and subsequent re-polyploidization to enable liver regeneration [8]. Together, these studies support that hepatocytes across the lobule mediate liver homeostasis and repair, rather than a locally confined liver stem cell population. However, sparse random labelling of hepatocytes or lineage tracing of subsets of hepatocytes marked by individual genes may not provide the full picture of when and where hepatocytes in different zones proliferate (Figure 1). Continuous capturing of all proliferative events across the entire hepatocyte pool over time could help shedding additional light on these important questions.
